# Integrated Analysis of the 2022 SARS-CoV-2 Omicron Lineage Replacement Dynamics in Connecticut, US

**DOI:** 10.3390/v17071020

**Published:** 2025-07-21

**Authors:** Nicholas F. G. Chen, Kien Pham, Chrispin Chaguza, Rafael Lopes, Fayette Klaassen, Chaney C. Kalinich, Yale SARS-CoV-2 Genomic Surveillance Initiative, Nicholas Kerantzas, Sameer Pandya, David Ferguson, Wade Schulz, Daniel M. Weinberger, Virginia E. Pitzer, Joshua L. Warren, Nathan D. Grubaugh, Anne M. Hahn

**Affiliations:** 1 Department of Epidemiology of Microbial Diseases, Yale School of Public Health, New Haven, CT 06510, USA; nicholas.chen@yale.edu (N.F.G.C.); nathan.grubaugh@yale.edu (N.D.G.); 2 Public Health Modeling Unit, Yale School of Public Health, New Haven, CT 06510, USA; 3 Department of Global Health & Population, Harvard T. H. Chan School of Public Health, Boston, MA 02115, USA; 4 Department of Laboratory Medicine, Yale School of Medicine, New Haven, CT 06510, USA; 5 Center for Computational Health, Center for Outcomes Research & Evaluation (CORE), Yale School of Medicine, New Haven, CT 06510, USA; 6 Department of Biostatistics, Yale School of Public Health, New Haven, CT 06511, USA; 7 Department of Ecology and Evolutionary Biology, Yale University, New Haven, CT 06511, USA; 8 Department of Microbiology and Immunology, University of Melbourne, at the Peter Doherty Institute for Infection and Immunity, Melbourne, VIC 3000, Australia

**Keywords:** variant fitness, Genomic surveillance, viral evolution

## Abstract

In 2022, consecutive sweeps of highly transmissible SARS-CoV-2 Omicron-derived lineages (B.1.1.529*) maintained viral transmission despite extensive antigen exposure from both vaccinations and infections. To better understand Omicron variant emergence in the context of the dynamic fitness landscape of 2022, we aimed to explore putative drivers behind SARS-CoV-2 lineage replacements. Variant fitness is determined through its ability to either outrun previously dominant lineages or more efficiently circumvent host immune responses to previous infections and vaccinations. By analyzing data collected through our local genomic surveillance program from Connecticut, USA, we compared emerging Omicron lineages’ growth rates, estimated infections, effective reproductive rates, average viral copy numbers, and likelihood for causing infections in recently vaccinated individuals. We find that newly emerging Omicron lineages outcompeted dominant lineages through a combination of enhanced viral shedding or advanced immune escape depending on the population-level exposure state. This analysis integrates individual-level sequencing data with demographic, vaccination, laboratory, and epidemiological data and provides further insights into host–pathogen dynamics beyond public aggregate data.

## 1. Introduction

Capacities for genomic surveillance saw an unprecedented rise during the COVID-19 pandemic [1]. The ability to closely monitor SARS-CoV-2 populations in real-time was crucial to discover and track SARS-CoV-2 variants of concern (VOC). The first VOC, Alpha (B.1.1.7) [2,3], emerged towards the end of 2020 and was followed in 2021 by Beta (B.1.351) [4], Gamma (P.1) [5], and Delta (B.1.617) [6]. In late 2021, the highly divergent Omicron (B.1.1.529) lineage [7] rapidly displaced Delta globally.

In 2022, SARS-CoV-2 transmission remained high all year, which stands in contrast to the summer lows observed during 2020 and 2021. This was characterized by consecutive sweeps of multiple Omicron lineages, with BA.1 (B.1.1.529.1) being the first lineage named as VOC Omicron, which shared a common ancestor with the D614G background in late 2020. This ancestor diverged into at least five main sublineages (BA.1-BA.5) [8,9]. The exact origin and nature of the emergence of these Omicron lineages remains unclear but several Omicron lineages did successfully spread worldwide [9] and second- and third-generation Omicron variants remain the dominant SARS-CoV-2 lineages to date.

Thus, 2022 saw a unique encounter between a novel, highly divergent variant of the SARS-CoV-2 virus and a host population that, through intensive vaccination campaigns and vaccine roll-out in many parts of the world, had built up significant amounts of immunity towards the original Wuhan-Hu-1 SARS-CoV-2. In this study, we aimed to reconstruct key lineage replacement dynamics of Omicron lineages exemplified in a well-monitored study setting in Connecticut, United States (US). For this, we drew upon sample data collected from a hospital-based surveillance system at the Yale New Haven Hospital (YNHH) through the Yale Genomic Surveillance Initiative from January 2022 to January 2023. Whole genome sequencing data together with relevant laboratory and individual-level metadata were available for approximately 5–10% of total reported cases across the state and the entire length of the study period, including outpatients and asymptomatic individuals. To characterize competitive growth advantages of variants during their emergence periods and evaluate their fitness advantage over their immediate predecessors, we estimated growth rates of each incoming lineage, compared average virus copy number from nasal swabs, and calculated the risk for vaccine breakthrough infections as a metric for the ability to (re-)infect antigen-experienced hosts.

The synthesis of high-resolution epidemiological, genomic, and immunological data enables us to assess and compare the lineage turnover of each Omicron lineage. Exploring lineage replacement dynamics on a population level allows us to evaluate in vitro findings in real-world setting and identify key parameters defining variant fitness. Doing so, we show that in 2022, Omicron lineages outcompeted previously dominant strains through different mechanisms including enhanced transmissibility and/or advanced ability to (re-)infect immune hosts. Our study highlights the ability to derive key aspects of pathogen lineage fitness by analyzing individual-level data drawn from genomic surveillance efforts together with demographic and epidemiological data. Such frameworks will be particularly relevant for further monitoring SARS-CoV-2 in the post-pandemic phase and are easily transferable to other pathogens.

## 2. Materials and Methods

### 2.1. Ethics Statement

The Institutional Review Board from the Yale University Human Research Protection Program determined that the RT-qPCR testing and sequencing of de-identified remnant COVID-19 clinical samples obtained from clinical partners conducted in this study is not research involving human subjects (IRB Protocol ID: 2000028599).

### 2.2. Data Sources

#### 2.2.1. Sample Collection and Processing

The samples analyzed here were collected between December 2021 and January 2023. Samples collected between May and December 2021 were used as a reference. SARS-CoV-2 positive samples (nasal swabs in viral transport media) were collected through the Yale New Haven Hospital (YNHH) System as a part of routine inpatient and outpatient testing and sent to the Yale SARS-CoV-2 Genomic Surveillance Initiative. Using the MagMAX viral/pathogen nucleic acid isolation kit (Thermofisher, Waltham, MA, USA), nucleic acid was extracted from 300 μL of each clinical sample and eluted into 75 μL of elution buffer. Extracted nucleic acid was then tested using a “research use only” (RUO) RT-qPCR assay [10] for SARS-CoV-2 RNA. Libraries were prepared for sequencing using the Illumina COVIDSeq Test (RUO version) and quantified using the Qubit High Sensitivity dsDNA kit (Life Technologies-Invitrogen, Waltham, MA, USA). Negative controls were included for RNA extraction, cDNA synthesis, and amplicon generation.

Prepared libraries were sequenced at the Yale Center for Genomic Analysis on the Illumina NovaSeq with a 2 × 150 approach and at least 1 million reads per sample.

Adaptor sequences were trimmed, primer sequences masked, and reads were aligned to the Wuhan-Hu-1 reference genome (GenBank MN908937.3) using BWA-MEM v.0.7.15 [11]. Consensus genomes were called (simple majority > 60% frequency) using iVar v1.3.133 [12] and SAMtool v 1.7 [13]. When <20 reads were present at a site an ambiguous “N” was used, with negative controls consisting of ≥99% Ns. The Pangolin lineage assignment tool [14] was used for assigning viral lineages.

#### 2.2.2. Individual Metadata

We obtained additional metadata and vaccination records from the YNHH system and the Center for Outcomes Research and Evaluation (CORE) and matched these records to sequencing data through unique sample identifiers. Duplicate patient records or those with missing or inconsistent vaccination data were removed. This includes instances of repeat infections where only the first instance was retained. We determined vaccination status at time of infection by comparing the sample collection date to the patient’s vaccination record dates.

We then categorized vaccine breakthrough statuses with respect to both the number of vaccine doses received more than 14 days prior to the collection date and the timing of the most recent vaccination relative to a 5-month period. Patient vaccination statuses at time of infection were thus categorized as: infection without vaccination (0 doses), one dose vaccine breakthrough, two or more dose vaccine breakthrough greater than 5 months since the most recent vaccination, or two or more dose vaccine breakthrough within 5 months since the most recent vaccination.

#### 2.2.3. Population Vaccination Trends

We obtained data on vaccination trends in Connecticut from the Centers for Disease Control and Prevention (CDC) [15].

#### 2.2.4. Population Variant Trends

We obtained variant trend data for Connecticut from the Global Initiative on Sharing All Influenza Data (GISAID) [16].

#### 2.2.5. Variant R_t_ and Immunity Estimates

We obtained variant cumulative estimated case counts from covidestim, a Bayesian nowcasting approach that incorporates reported cases, hospitalizations, immunity’ [17,18], exposures, and vaccination data to generate state and county level estimates of variant specific infections [19]. To obtain variant-specific R_t_ estimates, we used the variant-specific infection estimates from covidestim with the EpiEstim R package v.2.4 [20].

### 2.3. Analyses

#### 2.3.1. Variant R_t_ Ratios

To compare R_t_ values between variants, we first selected 14-day periods when a new variant was emerging in the population. For each of these time periods, we then divided the daily R_t_ value of the emerging variant by the R_t_ value of the established variant for the same calendar day to calculate daily R_t_ ratios [21].

#### 2.3.2. Variant Emergence Periods and Logistic Growth Rates

To determine the length of time each variant needed to become established in the population, we calculated daily frequencies for each variant across Connecticut using case count data from GISAID [16]. We then defined a variant’s emergence period as the date from when a variant first accounted for 5% of all cases to the first date the variant reached its maximum frequency in the population. We then fitted a locally estimated scatterplot smoothing (loess) curve to the data and extracted the fitted value corresponding to a frequency of 50% in the population. With this value we determined the number of days it took each variant to increase from a frequency of 5% in the population to 50%.

Using the same emergence periods, we fitted binomial logistic regression models where the variant lineage was modeled as a function of calendar time. For each model, the variant(s) being displaced in the population served as the reference group. We then extracted the coefficient of the predictor variable of each model to determine the logistic growth rate for each variant.

#### 2.3.3. Variant Ct Values over Time and in Periods of Emergence

To understand how Ct values change across time, we subset each variant to the period of time where it was above 10% frequency in the population using RT-qPCR data from the Yale SARS-CoV-2 Genomic Surveillance Initiative. For each of these time periods and variants we tested for heteroscedasticity via a Breusch-Pagan test, checked for non-linearity or outlier values by plotting the residual values against the fitted values, and tested for normality via a Q-Q plot and frequency histogram of model residuals. We then fitted linear regression models where the Ct value was modeled as a function of calendar time.

To compare Ct values between variants, we identified 3, 4-week periods of variant co-circulation using the same dataset. For the two periods of pairwise comparisons, we performed Wilcoxon rank sum tests. For the comparison between three variants, we performed a Welch’s ANOVA test as well as a one-way ANOVA test to test for concordance, followed by post hoc pairwise tests via Tukey’s honest significance test.

#### 2.3.4. Mixed Effect Multivariable Logistic Regression Models

To determine the impact of vaccinations in periods of variant emergence, we used sequencing data from the Yale SARS-CoV-2 Genomic Surveillance Initiative matched to vaccination data from the YNHH System and CORE to identify five, five-week periods of variant co-circulation. We selected the specific date ranges of these periods so as to balance the number of unvaccinated individuals attributed to each variant in each period.

For these five periods we then fit mixed effect multivariable logistic regression models with a dichotomous outcome of the co-circulating variants found in each time period. To dichotomize the outcome in periods when more than two variants were circulating, we aggregated variants that emerged or were displaced contemporaneously, with the reference set as the variant(s) that was being displaced in the population.

Model covariates were selected via an Akaike information criterion (AIC) selection criteria test and included vaccination status at the time of infection, patient sex (male or female), age (5–17, 18–39, 40–64, 65+), town of residence as a random effect, and calendar time as a linear predictor. Infections without vaccinations, male sex, and the 18–39 age group served as variable reference levels. Due to the inability for under 5 years olds to receive vaccinations for the majority of our study period, we restricted our analysis to individuals 5 years and older.

To test the impact of the date interval lengths, we performed a sensitivity analysis by modifying the period of emergence from 5 weeks to 3, 4, 6, 7, and 8 weeks and found minimal differences in the results.

#### 2.3.5. Factors Impacting XBB.1.5 Ct Values

To investigate the factors that impact Ct values associated with the XBB.1 variant infections, we subset sequencing data from the Yale SARS-CoV-2 Genomic Surveillance Initiative to only those infections caused by lineages within the XBB.1 parent lineage. We then identified calendar time, sex (female or male), patient class (inpatient, outpatient, or emergency), age (<18, 18–49, 50–69, 70+), and vaccination status at time of infection as variables that could impact Ct values.

To understand the impact of calendar time, we tested for heteroscedasticity via a Breusch-Pagan test, checked for non-linearity or outlier values by plotting the residual values against the fitted values, and tested for normality via a Q-Q plot and frequency histogram of model residuals. We then fit a linear regression model to the data with Ct value as a function of calendar time.

For the remaining variables we assessed the normality of the Ct value distributions via Shapiro–Wilk tests and the variance of the Ct value distributions via Bartlett or F-tests. To test for differences in Ct values by sex, we performed equal variance t-tests. For the remaining variables, we performed Welch’s ANOVA tests as well as one-way ANOVA tests to test for concordance, followed by post hoc pairwise comparisons via Tukey’s honest significance test.

#### 2.3.6. Conceptual SIR Model

To synthesize our findings, we constructed a conceptual framework of variant fitness based on the traditional SIR transmission model. As a conceptual framework, this model does not attempt to explicitly simulate transmission through quantitative approaches. Rather, the model provides a visual representation of the proposed mechanisms by which the Omicron lineages may have gained selective advantages based on the results of our other analyses and findings from the literature.

#### 2.3.7. Statistical Analysis and Data Availability

We used the R statistical software (v. 4.2.1) [22] for all statistical analysis and figures. Data and code used in this study are publicly available on GitHub (https://github.com/NickChen10/Omicron_project, accessed on 1 July 2025).

## 3. Results

### 3.1. Continuous Omicron Lineage Replacement Causes High Levels of Community Transmission Throughout 2022

**Figure 1:**
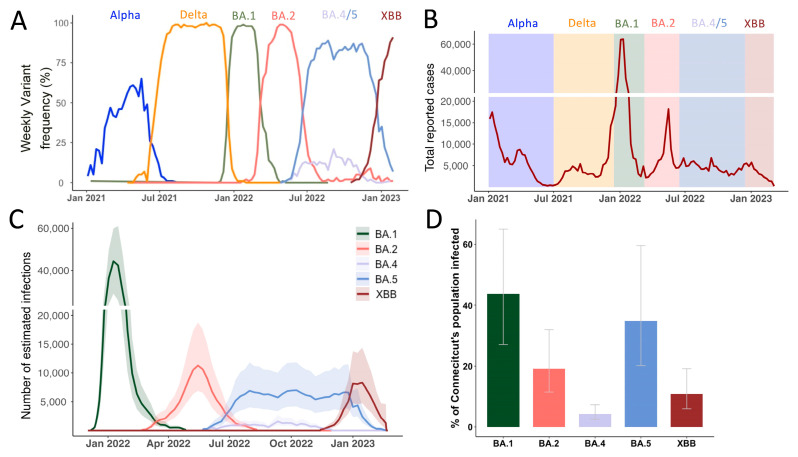
Genomic Epidemiology of SARS-CoV-2 describes the COVID-19 epidemic in Connecticut, US. (**A**) Frequencies of SARS-CoV-2 variants of concern from January 2021 to January 2023, based on sequences deposited on GISAID. (**B**) Reported COVID-19 infections from January 2021 to 2023 with highlights of the dominant periods of different Omicron lineages, data from the Connecticut Department of Public Health (**C**) Number of estimated infections based on the covidestim model for 2022 where testing and reporting widely changed after the first wave of Omicron BA.1 (**D**) Cumulative estimated cases for each Omicron variant up until January 2023.

Close monitoring of the SARS-CoV-2 population composition added valuable insights on how emerging variants were impacting COVID-19 case dynamics. The first VOCs detected in Connecticut were Alpha in late 2020 and Delta around mid-2021 (Figure 1A) and both introductions of VOCs were followed by a surge in reported SARS-CoV-2 cases in December 2020 and Spring 2021, respectively (Figure 1B). After a period of relatively low transmission in 2021 between May and September, cases started to rise again towards the end of the year with a second Delta-dominated peak in December 2021.

Globally, the first Omicron sample was recognized as a novel variant in November 2021 and shortly after also detected in the US and Connecticut (Table 1). Omicron was first found to consist out of 5 major sublineages BA.1-5 where BA.1 is an outgroup to the BA.2/BA.4/BA.5 cluster. XBB arose later through recombination of two second-generation BA.2 lineages (BA.2.10 & BA.2.75) and first emerged in Southeast-Asia around August-September 2022 [23] (Supplementary Figure S1). Notably, whereas BA.1-BA.5 most likely originated from a common source [7,8], XBB derived from lineages that emerged through sustained transmission chains in 2022.

After BA.1’s emergence in Connecticut in November 2021, the rapid replacement of Delta was accompanied by a massive increase in reported cases. Then, BA.2 swiftly replaced BA.1 in March 2022. BA.4 and BA.5 were both introduced to Connecticut in May 2022 and co-circulated for eight months until December 2022 (Table 1). Lastly, XBB-based variants outcompeted BA.5 lineages towards the end of 2022. Interestingly, the New England region including our study site was the first where XBB.1.5 was widely circulated within the US [24].

To understand the factors that influenced the consecutive sweeps of Omicron lineages in Connecticut, we first characterized case counts across our one-year study period for each lineage. As the northern hemisphere entered the summer months of 2022 (April to May onwards), most pandemic response mechanisms and non-pharmaceutical measures were discontinued. Consequently, case reporting is expected to be less reliable in accurately mirroring true infection dynamics compared to earlier in the pandemic. To overcome biases due to lack of reporting, differential testing behavior, potentially reduced symptoms after repeat infection or vaccination, we retrieved infection data from covidestim, a Bayesian nowcasting model. The model estimates infection dynamics beyond reported case numbers by incorporating publicly available time series of COVID-19 case notifications, hospitalizations, and deaths, accounting for vaccine-induced immunity to estimate infections [19,25]. To derive variant-specific estimated infections, we partitioned the weekly estimates of COVID-19 infections in Connecticut based on state-wide variant frequency (Supplementary Figure S2) to calculate variant-specific infection estimates cumulatively and over time by incorporating the frequencies of the major Omicron lineages derived from state-wide genomic surveillance (Figure 1C) [25]. We estimated that the BA.1 lineage caused approximately 1.5 million infections in Connecticut, USA (Figure 1D), representing around 44% (Credible Interval (CrI) 27–65%) of the state’s population. Together, the BA.2, BA.4, and BA.5 lineages caused over 2.1 million combined infections (CrI 1.2–3.5 million) within nine months after the BA.1 surge (Figure 1D). Another surge driven by the XBB lineage and its descendants caused an estimated 0.4 million infections (CrI 0.2–0.7 million) up until January 2023. Taken together, SARS-CoV-2 infection dynamics showed a high ability of incoming Omicron variants to replace dominate strains in a highly pathogen exposed population. We thus wanted to closer examine each of the emergence periods in more detail.

### 3.2. Growth Advantage of Emerging Omicron Lineages Shrinks Towards the End of 2022

**Figure 2:**
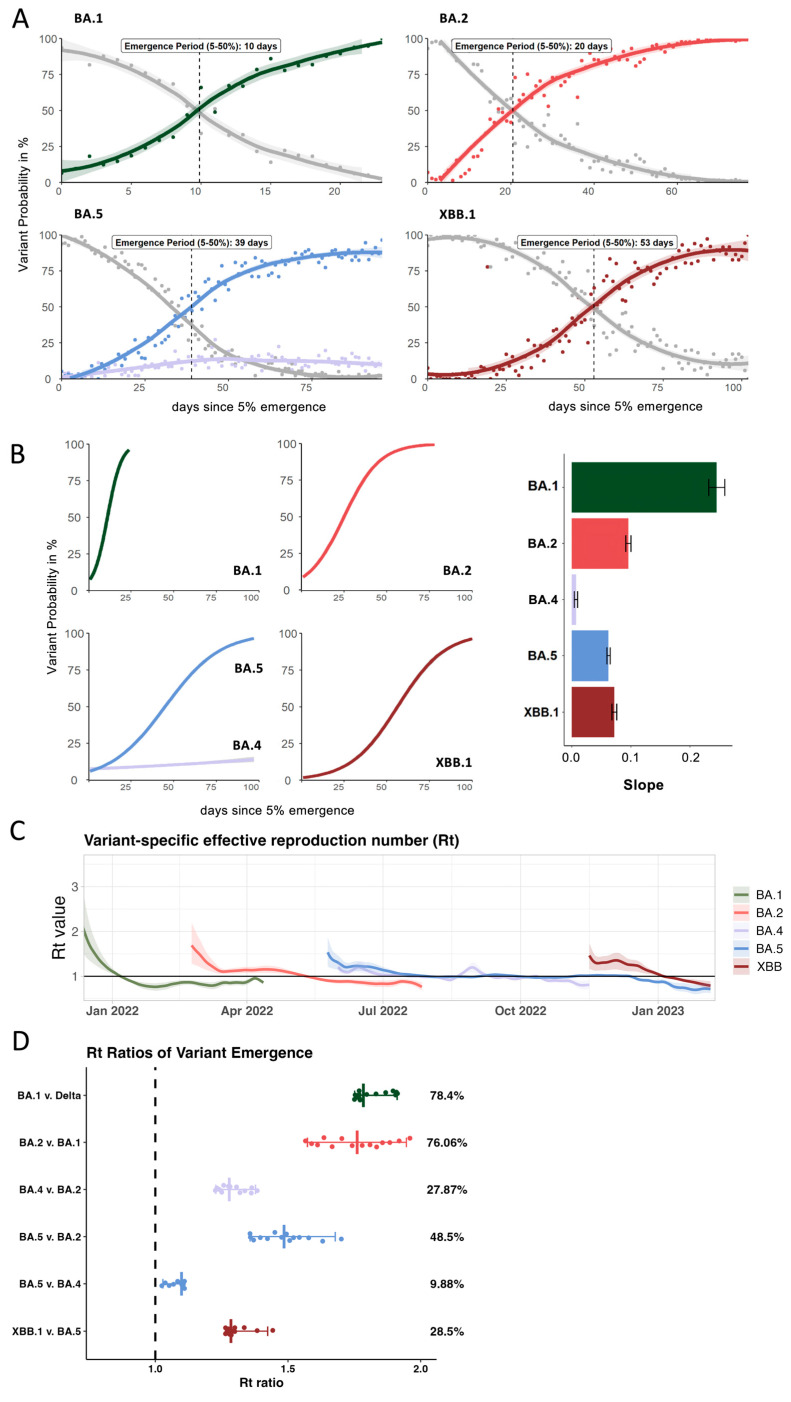
Comparison of key epidemiological parameters for each Omicron lineage in 2022. (**A**) Frequencies of the incoming Omicron lineage during its emergence period highlight the time interval between being detected at 5% and 50% of all sequences (based on submissions to GISAID for Connecticut). (**B**) Comparison of growth rates between the different Omicron lineages and the average slope of the growth curve during emergence periods (**C**) Variant-specific R_t_ numbers for each of the Omicron lineage over time derived from the modeled overall R_t_ from the covidestim model (**D**) Based on (**C**), the R_t_ ratios for each of the emergence periods were calculated to estimate the average advantage of each incoming lineage compared to the previously dominating one.

We determined the rate of lineage replacement by calculating the time it took for each incoming lineage to increase from 5% to 50% of reported daily cases in Connecticut (Figure 2A). This range was chosen to capture the period of time it took for a variant to become reliably established in the population, while avoiding some of the inherent stochasticity associated with initial cases. We then fitted a logistic regression model to the observed frequencies and compared their slope coefficients (Figure 2B). For BA.1, only 10 days passed between being detected at 5% to reaching 50% frequency (Figure 2A). For each of the subsequent lineages, lineage replacement slowed down, ranging from 20 to 59 days for BA.2 and XBB, respectively.

We further used the partitioned covidestim data to calculate variant-specific reproduction numbers (R_t_) over the course of 2022 (Figure 2C). We show that although BA.1 had the highest initial R_t_ value (2.4, CrI 1.6–3.5), it quickly dropped below R_t_ = 1 in early January. BA.2 emerged in February 2022 with an initial R_t_ of 1.7 (CrI 1.3–2.2), followed by a notable decrease and a plateau at around 1.2 for several weeks before falling below 1 around April 2022. The initial R_t_ of BA.5 (1.5 CrI 1.2–1.86) and BA.4 (1.2 CrI 1.07–1.36) were overall lower than for BA.1 and BA.2. However, BA.4 and BA.5 both hovered at R_t_ ~ 1 for several months during summer 2022. BA.4 was finally outcompeted by late-stage BA.5 lineages. The R_t_ for BA.5 only fell below 1 once XBB was introduced with an R_t_ of 1.4 (CrI 1.1–1.7). We observed similar initial R_t_ values for BA.5 and XBB and the introduction of XBB resulted in sustained levels of infections in absence of a distinct peak in cases (Figure 1C).

Based on previous work [21,26], we sought to understand the relative transmission advantages during the emergence periods by comparing R_t_ values of co-circulating lineages (Figure 2D). We first identified 14-day windows in which one lineage replaced the previously dominating lineage (Supplementary Table S1). We identified six, 14-day periods of variant overlap and divided the R_t_ value of the emerging lineage by the R_t_ value of the previously dominating lineage for each day of the emergence period, yielding 14 R_t_ ratios. The median of these values results in a comparative fitness measure (Figure 2D). We show that BA.1 was 78.4% more transmissible than Delta and BA.2 was 76% more transmissible than BA.1 (Figure 2D). The advantage of both BA.4 and BA.5 over BA.2 was significantly lower at 27.9% and 48.5%, respectively. While BA.5 had a slight fitness advantage of 10% over BA.4 during the emergence of both lineages, this advantage was not sufficient to fully outcompete BA.4 for several months. However, BA.4 was outcompeted by late-stage BA.5 sub-lineages such as BQ.1.1 and BF.7 (Table 1) before XBB emerged by late November. XBB replaced these late-stage BA.5 sequences with a fitness advantage of 28.5%. This reflects a 50% reduction in the transmission advantage of incoming XBB vs. the initial BA.5 lineages during BA.5 emergence, resulting in longer emergence period of ca. 55 days for XBB compared to 39 days for BA5.

Observing the variance in lineage replacement dynamics during the emergence windows for the major Omicron lineages, we next sought to further explore possible underlying drivers of the incoming lineages’ fitness advantages.

### 3.3. Average Inter- and Intra-Lineage Viral Copy Numbers Vary over Time Only Partially Explaining Lineage Replacement Patterns

**Figure 3:**
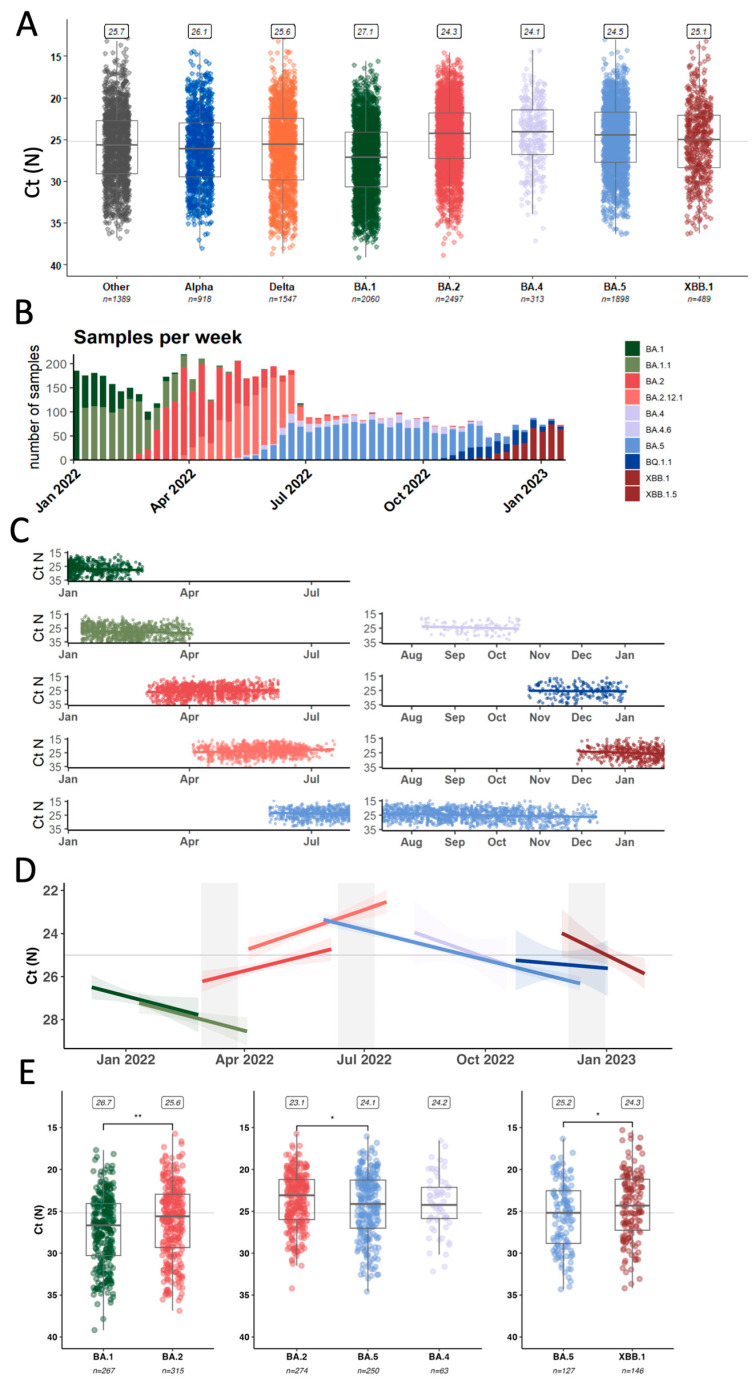
Comparing average qPCR Ct values as a proxy for variant intrinsic transmissibility. (**A**) Summary of all samples processed in our genomic surveillance program from 2021 to January 2023 (**B**) Overview about the numbers and distribution of Omicron samples (**C**) Ct values from Omicron samples plotted together with modeled average over time (**D**) Based on (**C**), summary values for each of the Omicron lineages with emergence periods highlighted (**E**) Statistical analysis of Ct values retrieved from samples collected during the emergence periods. * *p* < 0.05, ** *p* < 0.01.

To explain the specific mechanisms by which variants gain advantages over another, we evaluated the fitness advantage of each variant as a combination of intrinsic transmissibility and immune escape [27]. For this study, we used proxies derived from data collected through our genomic surveillance program for each of these factors. As a proxy for intrinsic transmissibility, we measured viral loads in nasopharyngeal swab material using a nucleocapsid (N)-based RT-qPCR assay [10]. We collected paired cycle threshold (Ct) and variant information across a total of 11,111 samples which enables us to compare average Ct and genome equivalents values per variant (Figure 3A, Supplementary Figure S3).

For further analyzing the drivers of lineage replacement in 2022, we focused on 6856 samples identified as Omicron-lineages (Figure 3B) collected from a wide range of individuals and disease statuses, including asymptomatic individuals detected through baseline surveillance of outpatients as well as inpatients and emergency department visits. Based on work from Hay et al. [28], we were particularly interested in the temporal resolution of Ct-value trends in our dataset (Figure 3C). The data was tested for the assumptions of linearity and fit to linear regressions (Figure 3D) to derive coefficients as a measure of Ct-value change over time for each lineage (Table 2).

Looking at trends over time, we noticed that the average Ct value for BA.1, BA.4, BA.5, and XBB samples increased over time (decreasing viral copy numbers) towards the end of each wave with a rate around 0.015–0.03 Ct values/day (Table 2). However, for BA.2 and BA.2.12.1 samples, we observed a decreasing trend towards the end of the BA.2 wave with a decrease of 0.015 and 0.021 Ct values/day.

Next, we compared only samples that were collected specifically during the emergence period of an incoming lineage, defined as 14 days on either side of the date where the previously dominant and the incoming variant each account for 50% of the samples (Supplementary Table S2).

Early BA.2 samples (n = 267) had significantly lower Ct values (median 25.6, Interquartile Range (IQR) 23.0–29.3) compared to late BA.1 samples (n = 315) (median 26.7, IQR 24.1–30.3) (*p*-value: 0.003) (Figure 3E). This supports previous reports from two European surveillance programs that also showed higher viral copies of BA.2 over BA.1 as measured by qPCR [29,30]. When looking at the emergence period of BA.4 and BA.5 compared to late-stage BA.2 samples, samples infected with the incoming variant had significantly higher Ct values than BA.2 samples (BA.4, n = 63,median 24.2, IQR 22.1–25,9; BA.5 n = 250, median 24.1, IQR 21.3–27.0, BA.2 n = 274, median 23.1, IQR 21.2–26.0) from that same period (*p*-value: 0.034). There was no statistically significant difference in the Ct values between BA.4 and BA.5 samples (*p*-value: 0.829). Finally, early XBB samples (n = 146) again had lower Ct values (median 24.3, IQR 21.2–27.3) compared to late-stage BA.5 samples (including BQ.1.1, n = 127) (median 25.2, IQR 22.5–28.8) (*p*-value: 0.033).

We also sought to investigate whether there was any association of individual metadata and Ct value in these first XBB cases by stratifying the samples according to age, sex, vaccination status, and patient class (Supplementary Figure S4) [31]. There was no significant difference in Ct values based on vaccination status. This is in line with previous findings from another cohort, where viral load dynamics did not differ significantly based on vaccination status once a productive infection was established [32]. However, we found that people admitted to the emergency department had, on average, lower Ct values compared to those tested as inpatients or outpatients.

In summary, a higher average viral copy number per variant was not necessarily decisive for a lineage’s fitness advantage as, for example, incoming BA.5 samples had on average lower viral copy numbers than late BA.2 samples. Interestingly, early cases of the incoming BA.2-derived XBB did exhibit higher viral loads than late BA.5 samples, mirroring the BA.1 to BA.2 transition. Thus, higher average viral loads could only partially explain a lineage success and we next assessed the ability of incoming Omicron lineages to circumvent previously generated immunity.

### 3.4. Likelihood of Incoming Lineages to Cause Breakthrough Infections in Recent Vaccinees Varies Across Different Omicron Lineages

**Figure 4:**
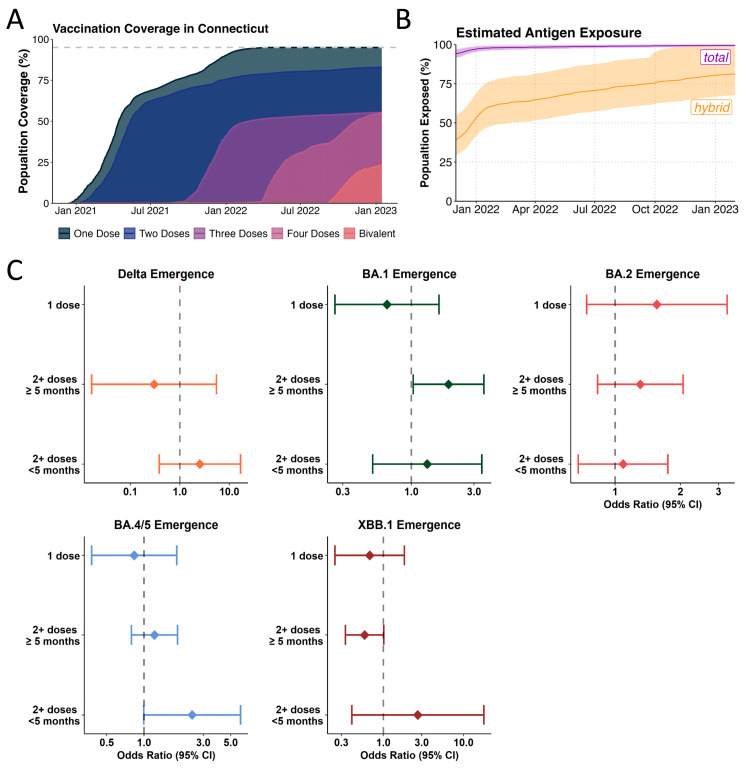
Influence of vaccine uptake and community-immunity levels on Omicron lineage emergence. (**A**) Vaccination coverage of Connecticut as reported by the US CDC for 2021 and 2022 (**B**) Estimated antigen exposure based on estimates from covidestim model according to either total (vaccination and/or infection) (purple) and hybrid exposure (infection and/or vaccination) (orange) (**C**) Logistic regression model to compare the ratios of infections caused by the incoming or previously dominant variant (Delta vs. pre-Delta variants (orange), BA.1 vs. Delta (green), BA.2 vs. BA.1 (pink), BA.4/5 vs. BA.2 (blue), XBB.1 vs. BA5 (dark red)) based on vaccination status during the emergence windows. BA.4 and BA.5 were analyzed together due to the similarity in the Spike region.

A blood donor seroprevalence survey showed that in December 2021, 95.5% of donors (95% Confidence Interval (CI) 93.5–96.9%) had antibodies against the Spike antigen, compared to 17.8% (CI 15.3–20.5%) with infection-induced antibodies [22,23,33,34]. The US CDC reports high vaccination coverage in Connecticut (70% coverage with 2 doses in September 2021) as well as uptake of a third dose booster towards the end of 2021 (55% in January 2022) (Figure 4A). Further, bi-valent booster shots (Wuhan-Hu-1 + BA.5) were administered to 23% of the population (Figure 4A). During 2022, the estimated proportion of individuals with at least one previous SARS-CoV-2 infection passed 75% in July [17,18] (Figure 4B). Thus, the pool of antigen-naïve hosts was greatly depleted towards mid-2022. Consequently, the fitness advantage of variants being able to infect hosts with previous immunity were expected to increase over time (Figure 4B). Accordingly, in experimental assays with serum collected from curated cohorts with defined antigen exposures, Omicron lineages BA.1, BA.2, BA.5 and XBB revealed sequential dips in sensitivity to neutralization by vaccine- and infection induced antibodies [35,36,37,38].

Both, immune escape and immune waning are expected to play a role in shaping variant fitness in a highly antigen-experienced population. To examine how the ability to infect antigen-experienced hosts contributed to the fitness advantages of incoming Omicron lineages, we compared the likelihood of the incoming lineage vs. dominant lineage to cause vaccine breakthrough infections (BTI) during the five-week emergence window (Supplementary Table S3). For this, we fitted mixed-effect multivariable logistic regression models adjusted for several potential confounding variables, including vaccination status, sex, age, location, and calendar time in a dataset of 14,246 individuals (Supplementary Table S4).

We show an increased odds of being infected with BA.1 (n = 414) compared to Delta (n = 446) amongst those vaccinated with at least 2 doses and who were more than 5 months from their most recent vaccination (OR: 1.92, 95% CI: 1.03–3.56) (Figure 4C, Supplementary Table S6). Thus, enhanced immune escape played a role in the emergence of BA.1 while not being a significant driver of fitness advantage in the emergence of Delta in 2021 (Supplementary Table S5). This result is in line with our previous study based on a similar study population that found enhanced odds of being infected with Omicron BA.1 in triple-vaccinated individuals [39]. Further, the lack of vaccine effects in the emergence of BA.2 (Supplementary Table S7) can be explained by the low antigenic distance between BA.2 and BA.1, especially in people having received 3 monovalent doses [40,41,42,43,44].

For the emergence of BA.2 (n = 391) over BA.1 (n = 314), we did not find overrepresentation in causing breakthrough infections during its emergence period, likely suggesting little to no effect of enhanced immune escape in our model (Figure 4C). During the emergence of BA.5 (n = 355), we found a tendency for an increased risk of having been infected with BA.5 vs. BA.2 (n = 319) when having been vaccinated in the last 5 months prior to infection (OR: 2.441, 95% CI: 0.995–5.988), though this did not reach statistical significance (Figure 4C, Supplementary Table S8). Several vaccine cohort studies have shown that BA.5 is more immune-evasive than BA.2 [45]. The lack of statistical significance seen for the emergence of BA.5 in our analysis may be attributable to the comparatively small sample size for these strata in our dataset (Supplementary Table S10) as well as putatively unreported, asymptomatic infections. Finally, for the emergence of XBB (n = 208), we found a tendency for XBB infections to be less likely among participants that are more than 5 months post most recent vaccination (OR: 0.586, 95% CI: 0.339–1.013) albeit this did not reach statistical significance in our analysis (Figure 4C, Supplementary Table S9).

Our analysis shows that the advantage an emerging lineage gained through circumventing previous immune responses varies over time and is increasingly difficult to analyze once infection and vaccination histories become more complex. Thus, we next explored how the infection dynamics and recorded vaccinations might have influenced the emergence subsequent variants.

### 3.5. Omicron Lineage Replacements in a Highly Antigen-Experienced Population

**Figure 5:**
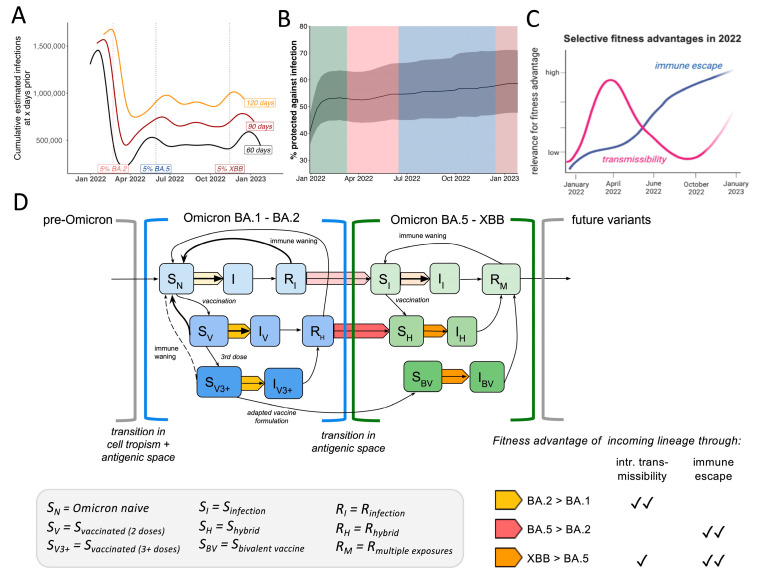
Summary of selective fitness advantages of Omicron lineage emergence in a dynamic host immunity landscape. (**A**) Sliding-window analysis of cumulative estimated infections 60, 90 or 120 days prior to each date on the plot with highlights when emerging Omicron lineages reached 5% of total samples (**B**) Frequency of estimated proportion (in%) of the population to be protected against infection over time inferred by vaccine and infection data according to the covidestim model (**C**) Conceptual visualization of selective advantages of incoming Omicron lineages over the year (**D**) SIR-based conceptual transmission model highlighting different fitness advantages of incoming Omicron lineages depending on the status of the host population. Shades of blue depict different levels of susceptibility within the first antigenic space (BA.1-BA.2) depending on infection and vaccination status where darker colors depict higher protection. Shades of green depict different levels of susceptibility within the second antigenic space (BA.5-XBB) depending on infection and vaccination status, darker colors depict higher levels of protection. Arrows in yellow, orange and red represent the different combinations of advantageous fitness traits as outlined in the table in the right corner. Shades of yellow, orange and red represent the strength of the transmission advantage effect applying to each transition.

To better understand the level of population-level immunity during variant emergence windows, we used covidestim infection numbers to calculate the number of recent infections 60, 90, or 120 days prior to the time point where an incoming lineage reached 5% of sequence frequency (Figure 5A). During BA.2 lineage emergence in March 2022, on a population-level, there were around 1.5 million BA.1 infections within the last 90 days, the time frame where antibody-mediated protection against re-infections is expected to remain high in the absence of substantial antigenic shifts. For the emergence of BA.5 and XBB, this figure was around 0.7 million. These high levels of infections gradually increased the proportion of individuals protected against reinfection as estimated by covidestim (Figure 5B) [17,18]. Based on these estimates, we schematically plotted the relative importance of either intrinsic transmissibility or antigenic distance for an emerging variant’s fitness advantage over the course of 2022 (Figure 5C). While immune escape increases gradually, following the number of people with Omicron antigen exposure, the advantage conferred through higher intrinsic transmissibility mainly played a role after the BA.1 and BA.5 waves, which infected up to 50% of the population each.

Lastly, we summarized our findings in a conceptual model based on a classical susceptible, infected, and recovered (SIR) compartmental dynamic transmission model highlighting fitness determinants of Omicron lineages (Figure 5D). Since Omicron BA.1 encompassed a vastly divergent Spike protein from pre-Omicron viruses, the previously observed high efficacy of vaccines, especially mRNA-platform based formulations, was greatly diminished [35,46,47,48]. In the model, this is reflected by opening a novel antigenic space (also defined as serotype) compared to previous pre-Omicron lineages [49]. We include three different levels of susceptibility depending on vaccine status (S_N_, S_V_, S_V3+_), as these strata have shown to harbor different quantities and qualities in antibody levels and thus differ in their capacity to mediate susceptibility against a first Omicron infection [50,51,52]. After the first BA.1 wave, 40–50% of Connecticut’s population moved from the ‘susceptible’ (S_N_ = Omicron-naive, i.e., no previous Omicron antigen exposure) compartment to ‘recovered’ (R_I_ for unvaccinated or R_H_ for vaccinated). The fraction of the population that just recovered from BA.1 (Figure 5A) was likely fairly well protected against a swift reinfection with either BA.1 or the antigenically similar BA.2 [53]. Based on epidemiological data, the transition from the R_I_/R_H_ bin back to the ‘susceptible’ bin within the same antigenic space is estimated to take around 6 months [54,55]. Incoming BA.2-derived lineages were competing with BA.1 to infect the remaining ‘susceptible’ population (i.e., Omicron-naive S_N_). Based on our analysis, we conclude BA.2 outcompeted BA.1 in reaching this susceptible population faster, causing higher viral loads in early-stage BA.2 infections but not overrepresentation among recent vaccinees (yellow arrow) (Figure 5D). After the BA.1 and BA.2 waves, approximately one-third of the population remained in the Omicron-naive compartment, limiting the fitness advantage of variants in the same antigenic space, even with higher transmissibility (like BA.2.12.1). Incoming BA.4 and BA.5 however were antigenically different and thus previous exposures to pre-Omicron or BA.1/BA.2 infections conferred less protective immunity, opened a second antigenic space (Figure 5D). Vaccinated or previously infected individuals transition from the ‘recovered’ bin in the previous antigenic space to the ‘susceptible’ (S_I_, S_H_) state in this second antigenic space (Figure 5D). Although BA.4 and BA.5 displayed lower intrinsic transmissibility compared to BA.2 (Figure 3D), these variants outcompeted BA.2 as they could spread in a larger fraction of people (the R_I_ & R_H_ bins) (red arrow) (Figure 5D). The continuously high and steady transmission levels of BA.4 and 5 during the northern hemisphere summer of 2022 (June-August) (Figure 1C) were likely defined by the rate of individuals moving from the recovered bin of the first antigen space to the S compartment in the second antigenic space depending on their recency of last antigen contacts (Figure 5D).

At the time XBB emerged, over 80% of the population had at least one Omicron antigen exposure (Figure 4B). In order to outcompete the ongoing BA.5 transmission chains (Figure 2D), incoming lineages had to maintain at least similar or higher levels of immune escape while also being more transmissible (orange arrow) (Figure 5D). Although XBB displays immune evasion from BA.5-induced immunity, some cross-neutralization, especially in individuals with previous Omicron antigen contact, was reported [56,57]. Thus, XBB outcompeted late-stage BA.5 samples through its extensive immune escape profile [58] paired with higher intrinsic transmissibility (Figure 3E) (orange arrow Figure 5D).

In summary, we examined real-world data collected through our local surveillance initiative combined with epidemiological modeling and genomic data from public databases to detailly describe lineage replacement dynamics. We revealed that lineage emergence was driven by factors like high intrinsic transmissibility or ability to escape previously generated immunity. These factors were distinctively distributed across the Omicron viral family. These insights help to explain and assess the risk of ongoing SARS-CoV-2 evolution well into the post-pandemic phase and underline the evolutionary flexibility of SARS-CoV-2 to exploring fitness niches in a highly immune host population.

## 4. Discussion

We evaluated the rapid lineage displacements of Omicron lineages in Connecticut during 2022. A key result from the data presented here is that SARS-CoV-2 fitness was not dictated by a single factor, but rather a fine-tuned combination of fitness advantages depending on previous population-level exposure histories [59].

We showed that in the beginning of 2022, BA.1 infected up to 40% of Connecticut’s population within a few weeks. Based on our estimates, we find that BA.2 fitness advantage was likely driven by higher viral loads and faster spread compared to late BA.1 infections. The emergence and continued spread of BA.4 and BA.5 early summer till November did not show higher viral copy numbers during the emergence window and thus was more likely driven by enhanced ability to circumvent previous immune responses and larger time spans between most recent infections and vaccinations. Towards the end of the year high levels of infection and vaccine-induced population immunity slowed down the growth rates of BA.5 and XBB.

While novel virus lineages are designated according to their phylogenetic identity, inferring epidemiological relevance from genetic data alone remains challenging. Being able to link genetic information with additional laboratory and epidemiological data of locally matched host–pathogen populations and their epidemic outcomes is crucial for evaluating the potential of novel variants [60]. We demonstrate the utility of an integrated approach for genomic surveillance data collection for identifying key characteristics of SARS-CoV-2 fitness dynamics with crucial data linkage to key demographic and laboratory metadata. This real-world, population-level analysis aligns well with previous work from laboratory studies and curated vaccinee cohorts [59]. To evaluate emerging lineages of known and unknown pathogens, such integrated approaches are needed for tailoring public health responses and informing vaccine formulations. Lessons learned from SARS-CoV-2 can be utilized and further adapted to other pathogens to maximize the usefulness of such surveillance programs for community health.

This retrospective analysis re-visits the lineage replacement dynamics of Omicron lineages that dominated Connecticut in 2022 and sheds light on potential underlying drivers of their sequential fitness advantages. This study exemplifies a use-case for combining sequencing data with epidemiological modeling and key laboratory data to better understand pathogen emergence.

## 5. Limitations

One limitation of this study might be the validity of these findings to other populations and settings. As outlined above, variant transmission advantages are intrinsically coupled to the local host population and thus our results are most applicable to other places with similar population characteristics, vaccination uptake rates, and public health policies. Whereas the specific dynamics of the different Omicron waves may vary locally, the data presented here is valuable for examining overall trends that are broadly applicable to other settings. Overall fitness advantages of each new Omicron lineage over their respective predecessor were fairly consistent globally [60].

As cross-sectional data has caveats and is sensitive to changes in epidemiological and viral factors [28,61] such as changes in tissue tropism, symptom severity, and test-seeking behavior are expected to differ for Omicron infections compared to infections with pre-Omicron lineages. Further, we are aware that Ct values do not necessarily scale with the amounts of infectious viral particles and that the kinetics of RNA clearance may not be directly related to clearance of infectious virus. However, our dataset for Ct value comparison is based on a pre-selection from the overall pool of available SARS-CoV-2 positive samples based on a Ct value cut-off in the diagnostic test that were expected to yield high sequencing coverage. We are usually able to isolate infectious virus from samples with Ct values below 30 (based on internal data) and thus estimate this cut-off reflects samples relevant for onward transmission and thus relevant for the purpose of this study. Additionally, we used a standard curve to convert Ct values to absolute viral genome copies and inter-plate variability. Further, symptom severity is thought to have remained similar across all Omicron lineages, reducing the potential for bias due to differences in test seeking behavior after the BA.1 emergence. Lastly, for the purposes of this study, we were interested in comparing trends between lineages in contrast to absolute values.

Lastly, in addition to co-circulating variants, the immune status of the population further plays a role in shaping R_t_ dynamics. For example, previous and recent exposures through infections and vaccinations together with the role-out of bivalent boosters starting in September 2022 (Figure 4A) might have influenced the slower XBB take-over dynamic compared to BA.5 (Figure 2A). To further address the influence of immunity from previous infections and vaccines on the transition dynamics of emerging lineages, would require more sophisticated, matched individual-level serological data which were not for the here described study cohort. Overall, waning of protection through vaccination and excess immune evasion will lead to either lower levels of neutralizing antibodies or reduced cross-reactivity of existing antibodies, leading to increased susceptibility even in the absence of novel variants but especially towards newer variants. Individual-level antibody profiling from patients in our cohort against a range of variants compared to time- and vaccine-status matched individuals that did not experience an infection from our catchment area could be used to attribute susceptibility to either waning antibody levels or antigen mismatch.

## Figures and Tables

**Table 1:** Major Omicron lineages in Connecticut January 2022–January 2023.

Lineage	First Detected Globally	First Detected in CT	Major Sub-Lineages in Connecticut (+Key Amino Acid Changes in Spike)
BA.1	October 2021	November 2021	BA.1.1 (+R346K)
BA.2	November 2021	January 2022	BA.2.12.1 (+L452Q, S704L)
BA.4	November 2021	May 2022	BA.4.6 (+R346T, N658S)
BA.5	November 2021	May 2022	BQ.1.1 (+R346T, K444T, N460K) BF.7 (+R346T)
XBB (BA.2.10*x BA.2.75*)	September 2022	October 2022	XBB.1.5 (+F486P)

* including sublineages.

**Table 2:** Trends in median Ct values of the major Omicron lineages over time.

Lineage	Median Ct Early Samples	Median Ct Late Samples	Regression Coefficient (Ct/Day) (*p*-Value)
BA.1	27.297 (IQR: 24.179–30.918)	27.799 (IQR: 26.139–28.760)	+0.016 (0.045)
BA.1.1	25.637 (IQR: 22.8 – 28.15)	28.399 (IQR: 25.563, 30–184)	+0.016 (0.011)
BA.2	26.681, IQR: 22.557–29.128	22.544 (IQR: 20.304, 25.718)	−0.015 (0.002)
BA.2.12.1	22.972 (IQR: 22.928–22.540)	23.335 (IQR: 22.928–22.540)	−0.021 (<0.001)
BA.4	21.2 (IQR: 18.944–23.75)	19.379 (IQR: 19.088–21.132)	+0.022 (0.227)
BA.5	23.077 (IQR: 19.61–25.606)	25.131 (IQR: 22.750–28.133)	+0.015 (<0.001)
BQ.1.1	23.231 (IQR: 21.580–24.561)	25.828 (IQR: 23.446–29.863)	+0.005 (0.758)
XBB	21.794 (IQR: 20.573–26.42)	24.753 (IQR: 19.846–27.566)	+0.03 (0.024)

## Data Availability

Data and code are publicly deposited on GitHub (https://github.com/NickChen10/Omicron_project, accessed on 1 July 2025).

## Supplementary Materials

The following supporting information can be downloaded at: https://www.mdpi.com/article/10.3390/v17071020/s1, Figure S1: Phylogenetic tree representing the genetic distance of different variants of concern as well as the Omicron lineages BA.1, BA.2, BA.4., BA.5 and XBB described in this study. The tree is based on a sub-sampling of 1000 samples primarily subsampled from the Yale-SARS-CoV-2 genomic surveillance initiative including some international reference sequences. The tree is rooted in the Wuhan-Hu1 sample. Across the Omicron family, a remarkable degree of convergent intra-lineage evolution has led to the emergence of at least one major sub-lineage. This is most clearly exemplified in the independent acquisition of amino acid changes at the same positions in the Spike region (e.g. BA.1.1 (R346K), BA.4.6 (R346T) and BQ.1.1 (R346T)); Figure S2: Yale SARS-CoV-2 Genomic Surveillance Initiative in 2022 (A) Frequency of % sequenced (red line) versus reported cases (grey bars) (B) Number and distribution of SARS-CoV-2 samples deposited on GISAID throughout 2022; Figure S3: Genome equivalents retrieved from qPCR assay qPCR Ct values were fitted to a standard curve to retrieve genome equivalents per ml; Figure S4: Analysis of viral loads measured in early XBB samples (A) Samples stratified according to sex, patient class, age and vaccination status (B) Raw data and plotted trend from XBB samples across all groups; Table S1: Emergence periods for growth advantage calculation; Table S2: Emergence periods for Ct value comparison; Table S3: Emergence periods for vaccine breakthrough infections comparison; Table S4: Patient demographics by Vaccination Status; Table S5: Delta Emergence Model; Table S6: BA.1 Emergence Model; Table S7: BA.2 Emergence Model; Table S8: BA.4/5 Emergence Model; Table S9: XBB.1.5 Emergence Model; Table S10: Vaccination Strata by Emergence Period.
