# Supplementary material for: Integrated Analysis of the 2022 SARS-CoV-2 Omicron Lineage Replacement Dynamics in Connecticut, US
Source: Viruses. 2025 Jul 21;17(7):1020. doi: 10.3390/v17071020 (PMC12299087; doi:10.3390/v17071020)
Supplement: Supplementary file 1 [file viruses-17-01020-s001.zip › viruses-3702590-supplementary.pdf]

Supplementary Figure S1

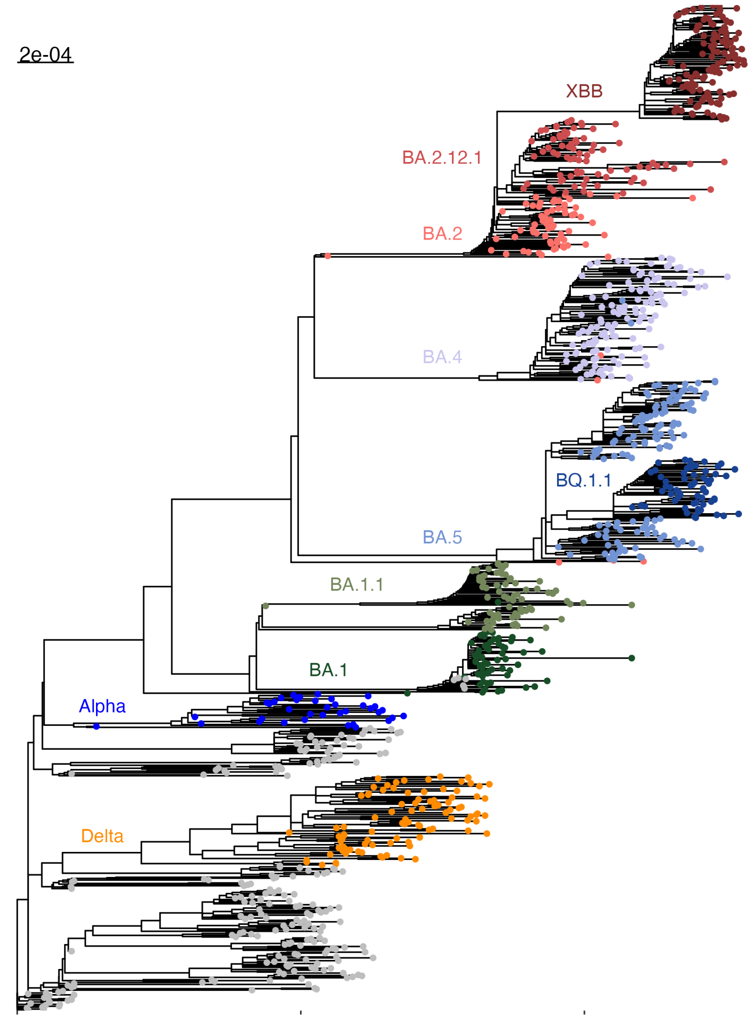

Figure S1 Phylogenetic tree representing the genetic distance of different variants of concern as well as the Omicron lineages BA.1, BA.2, BA.4., BA.5 and XBB described in this study. The tree is based on a sub-sampling of 1000 samples primarily subsampled from the Yale-SARS-CoV-2 genomic surveillance initiative including some international reference sequences. The tree is rooted in the Wuhan-Hu1 sample. Across the Omicron family, a remarkable degree of convergent intra-lineage evolution has led to the emergence of at least one major sub-lineage. This is most clearly exemplified in the independent acquisition of amino acid changes at the same positions in the Spike region (e.g. BA.1.1 (R346K), BA.4.6 (R346T) and BQ.1.1 (R346T)).

Supplementary Figure S2

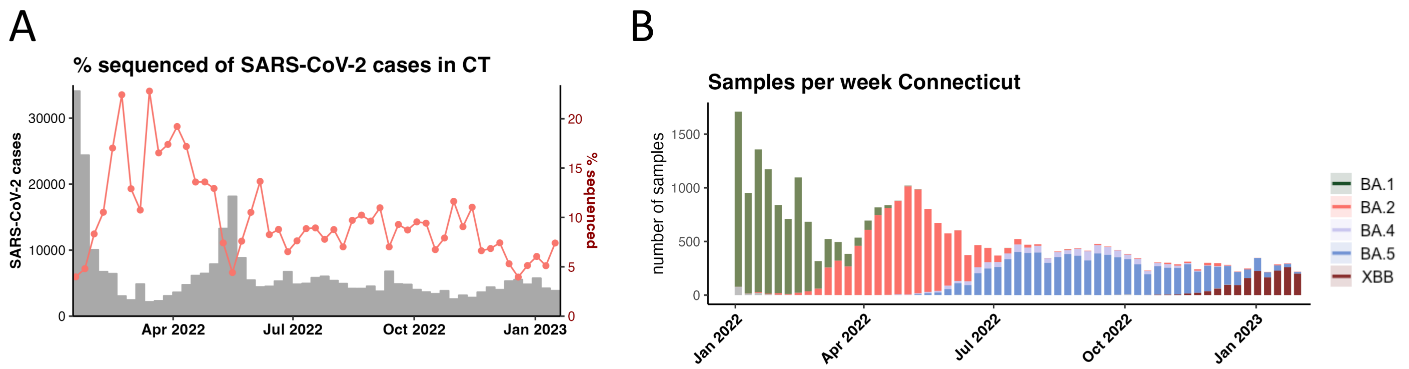

Figure S2 Yale SARS-CoV-2 Genomic Surveillance Initiative in 2022 (A) Frequency of % sequenced (red line) versus reported cases (grey bars) **(B)** Number and distribution of SARS-CoV-2 samples deposited on GISAID throughout 2022

Supplementary Figure S3

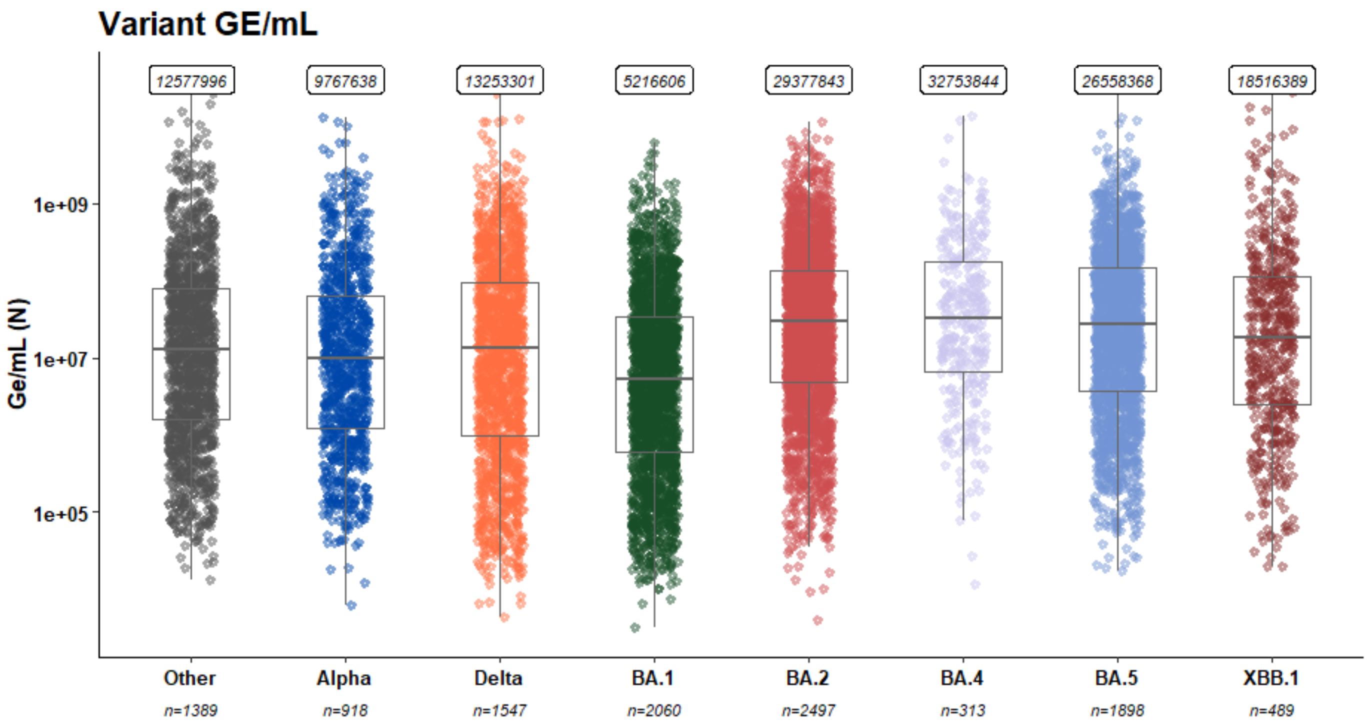

Figure S3 Genome equivalents retrieved from qPCR assay. qPCR Ct values were fitted to a standard curve to retrieve genome equivalents per ml.

Supplementary Figure S4

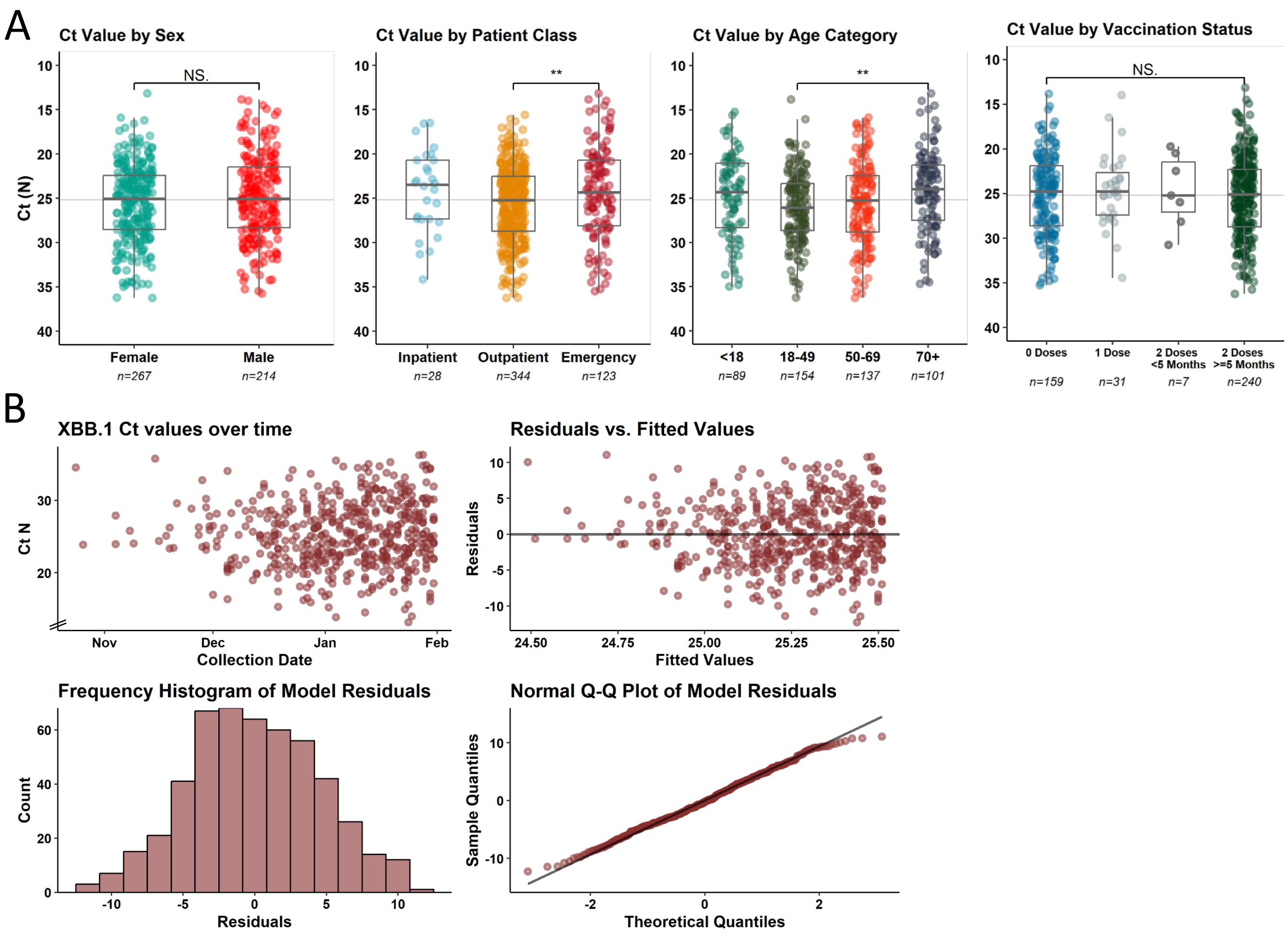

Figure S4 Analysis of viral loads measured in early XBB samples (A) Samples stratified according to sex, patient class, age and vaccination status **(B)** Raw data and plotted trend from XBB samples across all groups

Supplement

Supplementary Table S1 Emergence periods for Growth advantage calculation

Dominant Variant	Incoming Variant	Start	End
Delta	BA.1	Dec 15 2021	Dec 22 2021
BA.1	BA.2	March 6 2022	March 20 2022
BA.2	BA.4/5	June 18 2022	July 2 2022
BA.5	XBB	December 16 2022	December 30 2022

Supplementary Table S2 Emergence periods for Ct value comparison

Dominant Variant	Incoming Variant	Start	Crossover	End
BA.1	BA.2	February 27 2022	March 13 2022	March 27 2022
BA.2	BA.4/5	June 11 2022	June 25 2022	July 09 2022
BA.5	XBB	December 03 2022	December 17 2022	December 31 2022

Supplementary Table S3 Emergence periods for vaccine breakthrough infections comparison

Dominant Variant	Incoming Variant	Start	End
Delta	BA.1	November 30 2021	January 04 2022
BA.1	BA.2	February 23 2022	March 30 2022
BA.2	BA.4/5	June 09 2022	July 14 2022
BA.5	XBB	December 07 2022	January 11 2023

Supplementary Table S4: Patient demographics by Vaccination Status (n = 14246)

	Unvaccinated (n=7234)	1 dose (n=972)	2+ doses ≥ 5 months (n=4688)	2+ doses < 5 months (n=1352)	Overall (n=14246)
**Age group**					
5-17	2051 (28.4%)	49 (5.0%)	222 (4.7%)	124 (9.2%)	2446 (17.2%)
18-39	2635 (36.4%)	327 (33.6%)	1197 (25.5%)	403 (29.8%)	4562 (32.0%)
40-64	1999 (27.6%)	453 (46.6%)	2045 (43.6%)	558 (41.3%)	5055 (35.5%)
65+	549 (7.6%)	143 (14.7%)	1224 (26.1%)	267 (19.7%)	2183 (15.3%)
**Sex**					
Female	3273 (45.2%)	464 (47.7%)	2346 (50.0%)	743 (55.0%)	6826 (47.9%)
Male	3157 (43.6%)	413 (42.5%)	1744 (37.2%)	538 (39.8%)	5852 (41.1%)
Other	21 (0.3%)	1 (0.1%)	12 (0.3%)	0 (0%)	34 (0.2%)
Unknown	783 (10.8%)	94 (9.7%)	586 (12.5%)	71 (5.3%)	1534 (10.8%)

Supplementary Table S5: Delta Emergence Model

Covariates	Odds ratio (e^{β}, 95% CI)	p-value
**Vaccination status**		
Unvaccinated	Ref.	Ref.
One dose	NA	NA
2+ doses ≥ 5 months	0.302 (0.017, 5.481)	0.418
2+ doses< 5 months	2.533 (0.381, 16.818)	0.336
**Age category**		
18-39	Ref.	Ref.
5-17	0.573 (0.135, 2.444)	0.452
40-64	1.106 (0.296, 4.131)	0.881
65+	0.083 (0.008, 0.814)	0.033
**Sex**		
Male	Ref.	Ref.
Female	1.689 (0.562, 5.074)	0.350
**Time**	3.898 (2.023, 7.511)	<0.001

Supplementary Table S6: BA.1 Emergence Model

Covariates	Odds ratio (e^{β}, 95% CI)	p-value
**Vaccination status**		
Unvaccinated	Ref.	Ref.
One dose	0.651 (0.262, 1.621)	0.357
2+ doses ≥ 5 months	1.917 (1.033, 3.558)	0.0393
2+ doses< 5 months	1.320 (0.507, 3.440)	0.570
**Age category**		
18-39	Ref.	Ref.
5-17	0.280 (0.131, 0.598)	0.001
40-64	0.471 (0.258, 0.861)	0.014
65+	0.365 (0.146, 0.912)	0.031
**Sex**		
Male	Ref.	Ref.
Female	0.956 (0.574, 1.592)	0.863
**Time**	50.430 (27.761, 91.611)	<0.001

Supplementary Table S7: BA.2 Emergence Model

Covariates	Odds ratio (e^{β}, 95% CI)	p-value
**Vaccination status**		
Unvaccinated	Ref.	Ref.
One dose	1.558 (0.739, 3.285)	0.244
2+ doses ≥ 5 months	1.308 (0.830, 2.061)	0.247
2+ doses< 5 months	1.089 (0.677, 1.752)	0.724
**Age category**		
18-39	Ref.	Ref.
5-17	1.087 (0.614, 1.926)	0.775
40-64	1.003 (0.652, 1.542)	0.989
65+	1.251 (0.673, 2.327)	0.479
**Sex**		
Male	Ref.	Ref.
Female	1.066 (0.745, 1.526)	0.727
**Time**		<0.001

Supplementary Table S8: BA.4/5 Emergence Model

Covariates	Odds ratio (e^{β}, 95% CI)	p-value
**Vaccination status**		
Unvaccinated	Ref.	Ref.
One dose	0.834 (0.380, 1.835)	0.653
2+ doses ≥ 5 months	1.214 (0.791, 1.863)	0.374
2+ doses< 5 months	2.441 (0.995, 5.988)	0.051
**Age category**		
18-39	Ref.	Ref.
5-17	0.782 (0.396, 1.546)	0.480
40-64	0.858 (0.551, 1.335)	0.497
65+	0.630 (0.373, 1.062)	0.083
**Time**	2.924 (2.363, 3.617)	<0.001

Supplementary Table S9: XBB.1.5 Emergence Model

Covariates	Odds ratio (e^{β}, 95% CI)	p-value
**Vaccination status**		
Unvaccinated	Ref.	Ref.
One dose	0.677 (0.251, 1.830)	0.442
2+ doses ≥ 5 months	0.586 (0.339, 1.013)	0.056
2+ doses< 5 months	2.690 (0.405, 17.859)	0.306
**Age category**		
18-39	Ref.	Ref.
5-17	0.538 (0.218, 1.326)	0.178
40-64	0.575 (0.313, 1.056)	0.075
65+	0.539 (0.290, 0.999)	0.0497
**Sex**		
Male	Ref.	Ref.
Female	1.372 (0.865, 2.177)	0.179
**Time**	2.251 (1.765, 2.872)	<0.001

Supplementary Table S10: Vaccination Strata by Emergence Period

Emerging Variant	**Delta**	**BA.1**	**BA.2**	**BA.4/5**	**XBB**
**Vaccination Status**					
Unvaccinated	72	293	213	174	101
One dose	3	82	50	41	24
2+ doses ≥ 5 months	2	376	234	378	240
2+ doses< 5 months	9	78	181	36	6
Total	86	829	678	629	371
